# Diverse Clinical Phenotypes of *CASK*-Related Disorders and Multiple Functional Domains of CASK Protein

**DOI:** 10.3390/genes14081656

**Published:** 2023-08-20

**Authors:** Takuma Mori, Mengyun Zhou, Katsuhiko Tabuchi

**Affiliations:** 1 Department of Neuroinnovation, Institute for Biomedical Sciences, Interdisciplinary Cluster for Cutting Edge Research, Shinshu University, Matsumoto 390-8621, Japan; ktabuchi@shinshu-u.ac.jp; 2 Department of Molecular and Cellular Physiology, Shinshu University School of Medicine, Matsumoto 390-8621, Japan; 20hm124d@shinshu-u.ac.jp

**Keywords:** microcephaly with pontine and cerebellar hypoplasia (MICPCH), calcium/calmodulin-dependent serine protein kinase (CASK), *CASK*-related disorders, developmental epileptic encephalopathy, X chromosome inactivation, X-linked intellectual developmental disorders with nystagmus, neurocircuit interference

## Abstract

*CASK*-related disorders are a form of rare X-linked neurological diseases and most of the patients are females. They are characterized by several symptoms, including microcephaly with pontine and cerebellar hypoplasia (MICPCH), epilepsy, congenital nystagmus, and neurodevelopmental disorders. Whole-genome sequencing has identified various mutations, including nonsense and missense mutations, from patients with *CASK*-related disorders, revealing correlations between specific mutations and clinical phenotypes. Notably, missense mutations associated with epilepsy and intellectual disability were found throughout the whole region of the CASK protein, while missense mutations related to microcephaly and MICPCH were restricted in certain domains. To investigate the pathophysiology of *CASK*-related disorders, research groups have employed diverse methods, including the generation of *CASK* knockout mice and the supplementation of CASK to rescue the phenotypes. These approaches have yielded valuable insights into the identification of functional domains of the CASK protein associated with a specific phenotype. Additionally, recent advancements in the AI-based prediction of protein structure, such as AlphaFold2, and the application of genome-editing techniques to generate *CASK* mutant mice carrying missense mutations from patients with *CASK*-related disorders, allow us to understand the pathophysiology of *CASK*-related disorders in more depth and to develop novel therapeutic methods for the fundamental treatment of *CASK*-related disorders.

## 1. *CASK*-Related Disorders, X-Linked Neurological Disorders

Calcium/calmodulin-dependent serine protein kinase (CASK)-related disorders are a syndrome of multiple pathologies that occur mostly in the nervous system. The *CASK* gene is located on the X chromosome at Xp11.4 [1,2] and several clinical phenotypes of *CASK*-related disorders have been identified. Historically, Dimitratos et al. first reported the association between the *CASK* gene and the clinical phenotype in 1998 [2]. They reported that a deletion of Xp11.4, which contains the *CASK* gene, was found in patients with X-linked optic atrophy. In 2007, a case of a female patient with a relatively large genomic deletion containing the *CASK* gene was reported by Froyen et al. [3]. This patient exhibited microcephaly as well as intellectual disability, suggesting that the *CASK* gene may be involved in X-linked intellectual disability (XLID). The genomic deletions described in this report include genes other than the *CASK* gene, and it was not concluded that the *CASK* gene was the cause of microcephaly and XLID. In 2008, Najm et al. further confirmed the association between *CASK* gene mutations and microcephaly with cerebellar and pontine hypoplasia (MICPCH) and XLID in five patients [4]. Subsequently, epilepsy syndromes occurring in neonates and infants were also reported by several research groups [5,6,7,8,9,10]. To date, *CASK*-related disorders comprise various developmental disorders such as MICPCH [4,5], X-linked intellectual disability [7,11], FG syndrome [12,13], pediatric epilepsy syndrome [5,6,7,8,9,10], ophthalmologic phenotypes [13,14] (such as nystagmus), hearing impairment [5,7,11], and autism spectrum disorders [15]. Although CASK is known to be involved in other biological aspects such as cardiogenesis [16] and tumorigenesis [17,18,19,20], this review will focus on the biological role of CASK in the nervous system and the pathophysiology of *CASK*-related disorders.

## 2. Protein Structure and Protein Interaction of CASK

CASK was originally identified as an intracellular binding partner of neurexin, a synaptic adhesion molecule [21]. The *CASK* gene encodes a multiple-domain scaffolding protein, which consists of a catalytic Serine/Threonine Kinase (aka calmodulin kinase (CaMK)) domain, two Lin2/Lin7 (L27.1 and L27.2) domains, a PSD-95/discs large/ZO-1 (PDZ) domain, an Src Homology 3 (SH3) domain, and a Guanylate Kinase (GuK) domain, and each domain plays different roles through their interactions with other proteins [21,22,23].

**Figure 1:**
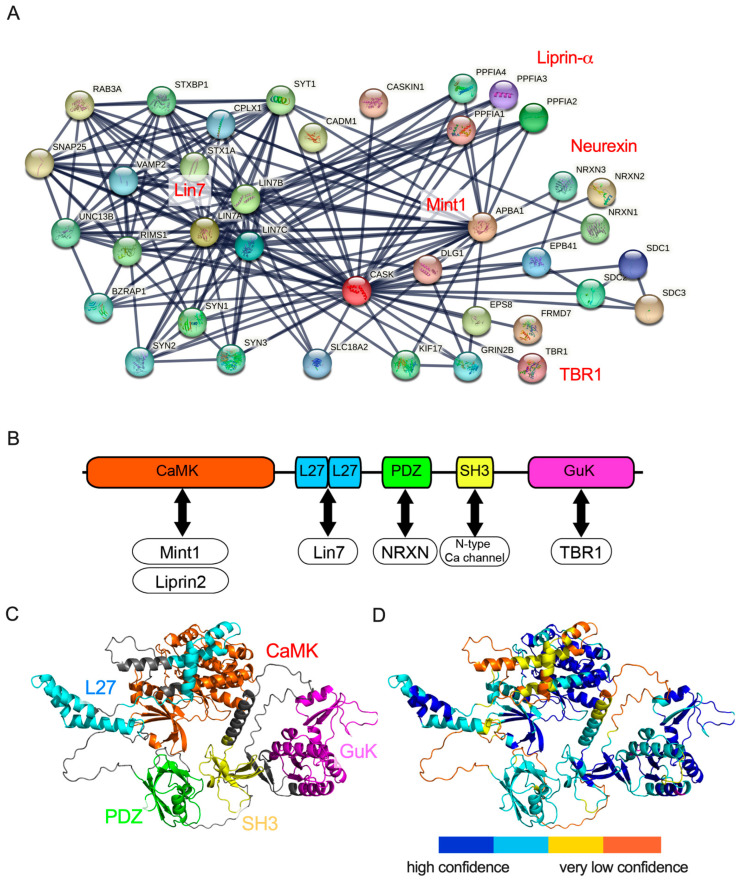
Protein–protein interaction network and predicted protein structure of CASK. (**A**) CASK-protein interaction network produced by String database [44]. Protein families that have been intensively investigated are indicated in red. (**B**) Functional domains of CASK and interactive partner molecules in (**A**). Five functional domains of CASK, CaMK, L27, PDZ, SH3, and GuK are colored orange, cyan, green, yellow, and magenta, respectively. (**C**) A three-dimensional structure of CASK is predicted by ColabFold, an AlphaFold2 on Google Colab [45]. (**D**) The confidence of the prediction is colored based on the score of pLDDT. High-confidence (pLDDT > 90) is drawn in blue and very low confidence is shown in yellow. The angle of the 3D structure of CASK is identical between C and D.

After neurexin was identified as a binding partner of CASK, more than 50 proteins were demonstrated to bind to CASK (Figure 1A). Biochemical studies have determined the functional domains of CASK recruited in protein–protein interactions (Figure 1B). The CaMK domain at the N-terminal of CASK functions as a magnesium-dependent atypical kinase, which could phosphorylate the C-terminal of neurexins [24]. The CaMK domain has also been demonstrated to interact with other molecules such as liprin-α and Mint1 [25,26,27,28,29]. The L27 domain interacts with the Lin-7 (Veli) family [22,23] and SAP-97 (DLG1) [27,30,31], a member of PSD-95-like membrane-associated guanylate kinases. Both lin-7 and SAP-97 bind to the L27 domains of CASK through its L27 domain on the N-terminal and C-terminal, respectively. SAP97 and Lin-7 are also scaffolding proteins and assemble synaptic proteins. CASK and SAP-97 have been shown to mediate the sorting of the N-methyl-D-aspartate (NMDA) receptor and CASK and Lin-7 regulate the localization of the inward rectifier potassium channel Kir2.3 on the basolateral membrane [32,33]. The PDZ domain of CASK has been shown to bind to trans-membrane proteins including neurexins and syndecans [1,34,35]. Neurexins are presynaptic adhesion proteins that bind to postsynaptic adhesion molecules such as neuroligins and the leucine-rich repeat trans-membrane neuronal proteins (LRRTMs) to construct and maintain the synaptic structure and function [36]. The SH3 domain of CASK binds to the proline-rich region at the carboxyl terminal of the N-type calcium channel and may regulate synaptic vesicle exocytosis at presynaptic axon terminals [37,38]. The GuK domain located at the C-terminal of CASK has been shown to bind to transcription factors, TBR-1, and CASK-interacting Nucleosome Assembly Protein (CINAP) [39,40,41]. The complex of CASK and TBR1 is trans-located in the nuclei of neurons and regulates the expression of genes such as *grin2b* and *reln*, which control neuronal migration and maturation [40,42,43].

These functional domains of CASK cooperatively bind to partner proteins, enabling CASK to carry out its biological functions. For instance, the PDZ domain of the CASK protein has been recognized as the primary binding domain for neurexin [1,34,35]. However, a recent report of two CASK missense mutations from patients with MICPCH suggested that not only the PDZ domain but also the SH3 and GuK domains of CASK may contribute to the binding between neurexin and CASK [46]. This indicates that the protein interactions between CASK and its partners could be established through multiple domains of the CASK protein.

To understand the macro-view of the protein–protein interaction, it is essential to gain information on the three-dimensional (3D) structures of CASK proteins. Until a few years ago, the 3D structures of each functional domain were demonstrated using X-ray crystallography and nuclear magnetic resonance (NMR) spectroscopy, and these data are widely available from the RCSB Protein Data Bank. However, the complete 3D structure of the entire CASK protein remained elusive. Recently, more sophisticated predictions of protein structures were made available using artificial intelligence-based programs, such as AlphaFold [47]. In our investigation, we used ColabFold [45], which is a web-based implementation of AlphaFold2 on Google Collaboratory, to predict the protein structure of CASK (Figure 1C). The prediction achieved high quality in the scale of each functional domain of the CASK protein. The prediction of the entire structure of the CASK protein, however, may not be accurate enough because the model’s confidence is not sufficiently high, especially in the linker regions bridging functional domains of the CASK protein (Figure 1D). Thus, the relative topology of functional domains of the CASK protein may not be precisely predicted by AlfphaFold2. Future improvements in the AI-based prediction will be required to advance our understanding of the biophysical mechanisms of the CASK–protein interactions and to explore potential novel partner proteins binding to CASK.

## 3. Genetics of *CASK*-Related Disorders

*CASK*-related disorders are caused by pathogenic variants in the *CASK* gene and include a spectrum of clinical phenotypes. *CASK*-related disorders are classified into two primary phenotypes, MICPCH, and XLID with or without nystagmus. Typically, MICPCH is associated with loss-of-function (i.e., nonsense or frameshift) mutations of the *CASK* gene, while XLID with or without nystagmus appears to be associated with hypomorphic (i.e., missense) mutations of the *CASK* gene. We first used the ClinVar database on NCBI [48] and found a total of 306 variants, consisting of 37 frameshift variants (two cases with substitution with a stop codon), 227 missense variants, and 43 nonsense variants (Table 1). Almost all the frameshift and nonsense variants are classified as pathogenic or likely pathogenic, whereas some of the missense variants are classified as benign, indicating that frameshift and nonsense mutations may cause a severer phenotype than missense mutations (*p* < 0.0001, Chi-square test). However, some variants are not associated with clinical phenotypes on the database (12/37 in frameshift; 58/227 in missense; and 15/44 in nonsense). To evaluate the association between the mutations of the *CASK* gene and clinical phenotypes, we investigated 49 reports describing 197 patients with *CASK*-related disorders [4,5,6,7,8,9,10,11,12,13,14,46,49,50,51,52,53,54,55,56,57,58,59,60,61,62,63,64,65,66,67,68,69,70,71,72,73,74,75,76,77,78,79,80,81,82,83,84,85] and classified the cases based on the genders and phenotypes (Table 2).

Intellectual disability (ID) is been commonly observed both in males (96.1%) and females (93.5%; *p* = 0.4315, males vs. females; statistically not significant between gender, Chi-square test) affected by *CASK*-related disorders. MICPCH is relatively common in patients with *CASK*-related disorders at a lower frequency, both in males (76.0%) and females (87.7%; *p* = 0.0634, males vs. females; statistically not significant between gender, Chi-square test). Another prominent phenotype associated with *CASK*-related disorders is epileptic syndromes, encompassing various types of epileptic seizures, including Ohtahara syndrome (aka epileptic encephalopathy with suppression burst of spikes), West syndrome (aka infantile spasms), absence epilepsy, myoclonic seizures, and focal seizures. Epileptic phenotypes were reported in males (54.1%) more frequently than in females (36.1%, *p* = 0.0198, males vs. females; statistically significant *p* < 0.05, Chi-square test). Giacomini et al. investigated epileptic phenotypes of 34 Italian patients with *CASK*-related disorders and reported that half of the patients (50%, 17/34) showed epileptic EEG patterns. Considering these findings together, it is evident that approximately half of the patients suffered from epileptic seizures.

**Figure 2:**
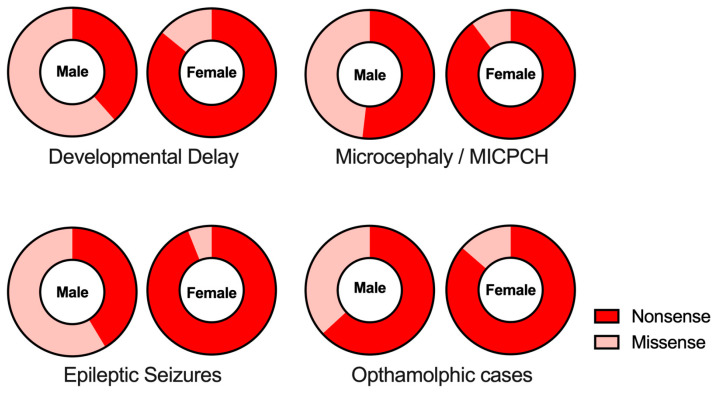
Gender-dependency of *CASK* mutation types in clinical phenotypes. The proportions of nonsense (red) and missense (pink) mutations found in clinical phenotypes are shown in each gender.

The relationship between clinical phenotypes and types of mutations in the *CASK* gene shows a sexual difference (Table 3 and Figure 2). Specifically, nonsense mutations, including frameshift mutations, are more prevalent in females than in males. In females, more than 85% of the cases are due to nonsense mutations of the *CASK* gene, whereas 38~63% of the cases are caused by nonsense mutations in males (*p* < 0.0001, intellectual disability; *p* < 0.0001, MICPCH; *p* < 0.0001, epilepsy; *p* = 0.0169, opthalmological anomalies; statistically significant in all cases, see Figure 2).

**Figure 3:**
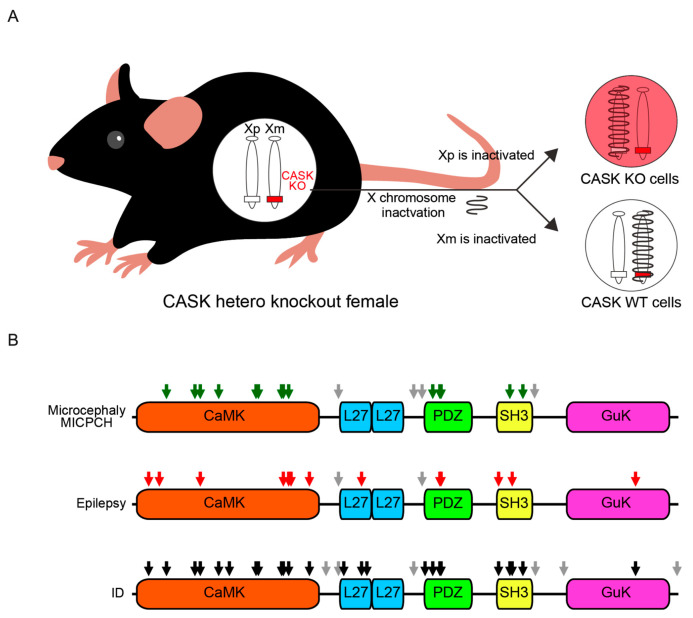
X chromosome inactivation of the *CASK* gene and the genomic locus of the missense mutations associated with specific clinical phenotypes. (**A**) A schematic drawing of X chromosome inactivation (XCI) of the *CASK* gene in *CASK*-related disorders. Female mice, as well as human patients, have two X chromosomes, and one of them carries a non-functional/hypomorphic *cask* gene. One of the two X chromosomes is inactivated and the cells with non-functional (red) and functional (white) *cask* are generated. (**B**) A schematic drawing of functional domains of CASK protein and the locations of missense mutations. Grey arrows represent missense mutations outside the five functional domains. Green arrows (top) are the missense mutation identified from patients with MICPCH, red arrows are those with epilepsy, and black arrows are those with ID.

In *CASK*-related disorders, nonsense and frameshift mutations are speculated to lead to loss-of-function variants, while missense mutations to hypomorphic variants. Although there are reports that missense or nonsense mutations could become gain-of-function in other disorders (e.g., NLGN3^R451C^ missense mutation [86,87], and SIK1^Q633X^ nonsense mutation [88,89]), to our knowledge, none of the nonsense and missense variants of the CASK protein were demonstrated to be gain-of-function or dominant-negative mutations according to biochemical analysis. The difference in effects between genders may be due to the regulation of the *CASK* gene by X chromosome inactivation (XCI) [90,91,92]. In males, a mutation in the X chromosome affects all somatic cells. In females, XCI silences one X chromosome, resulting in two cell types: those with CASK variants and those with normal CASK protein (Figure 3A). This could explain how normal cells in females mitigate the impact of loss-of-function CASK variants and how male patients with loss-of-function mutations exhibit severer symptoms, potentially leading to early lethality.

## 4. Phenotypes and Functional Domains of CASK

Most nonsense and frameshift mutations are considered to result in the degeneration of the protein, mainly by nonsense-mediated mRNA decay (NMD) [93]. NMD is one of the mRNA quality-control mechanisms of eukaryotes. When an immature stop codon is created in the translation region by the nonsense or frameshift mutations, the abnormal mRNA is degraded and almost no protein is translated. On the other hand, it is known that missense mutations can produce the full length of the protein with hypomorphic functions. This idea seems to be supported by the data of ClinVar, showing that more variants classified as the clinical significance of benign were observed in missense mutations (Table 1). Missense mutations generally disrupt the normal biological functions of a specific domain of the protein by changing the affinity of protein–protein interactions. Figure 3B shows the regions of missense mutations in the CASK protein found in patients, and the missense mutations associated with MICHPCH/microcephaly are confined to CaMK, PDZ, and SH3 domains, indicating that these domains play important roles in brain development, especially in the cerebellum.

MICPCH/microcephaly is one of the phenotypes of *CASK*-related disorders that has been extensively examined in animal models. The cerebellar size of *cask* heterozygous knockout female mice and hypomorphic male mice was shown to be smaller than that in wild-type mice [4,92,94,95]. Similarly, we reported that the knockout of neurexin, a main binding partner of CASK, from the cerebellar granule cells, induced cell death. The survival of cerebellar granule cells crucially depends on the presence of neurexin-1, -2, and -3 isoforms [96]. Intriguingly, in vitro studies demonstrated that the intracellular domain of neurexin, which includes the PDZ domain, was essential to the survival of cerebellar granule cells. The importance of the PDZ domain in cerebellar development is supported not only by genetic findings of missense mutations in the PDZ domain but also by our recent report using cerebellar granule cell culture. We demonstrated that the death of cerebellar granule cells from the conditional knockout of *CASK* can be rescued by the full length of CASK but not by the variants lacking the CaMK, PDZ, or SH3 domains of CASK, indicating that all three domains are essential for the survival of cerebellar granule cells in mice [95]. Moreover, one of the MICPCH-associated missense mutations (M519T), which could not bind to neurexin in vitro assay [63], failed to rescue the cell death of cerebellar granule cells from the knockout of the *cask* gene [95], indicating that CASK–neurexin binding would be important to cerebellar development. Although the molecular mechanism of cerebellar granule cell death is still unclear, one possible hypothesis is the neurexin-dependent release of brain-derived neurotrophic factor (BDNF) [96], which plays an essential role in cerebellar development in mice and primates [97,98,99].

In addition to the PDZ domain, other functional domains of the CASK protein are known to play important roles in binding CASK and neurexin. For instance, the CaMK domain of CASK does not include a known binding motif to neurexin but has been reported to bind to other proteins such as Liprin-α and Mint1 [25,37]. These molecules have been demonstrated to stabilize the binding between CASK and neurexin [100]. Thus, the CASK–neurexin–Liprin-α tripartite complex may be a molecular mechanism of the development of the cerebellum. Interestingly, a mutation in the CaMK domain, R106P, which was identified from a male patient of MICPCH [57], did not prevent the cell death of cerebellar granule cells [95]. The specific residue has been shown to contribute to interactions between CASK and Liprin-α and Mint1 [25], and an AlphaFold2-prediction of CaMK protein structure revealed that the mutation disrupted the interactions [95]. The contribution of CASK–neurexin interactions to cerebellar development is unclear, partially because limited information is available on the molecular structure between CASK and neurexins. More evidence on the molecular mechanism will help to understand that the interaction between CASK and neurexin plays an important role in the pathogenesis of MICPCH caused by *CASK* deficiency.

Another phenotype of *CASK*-related disorders is infantile epileptic syndrome, occurring in a smaller proportion of patients with *CASK*-related disorders (53.6% in males and 35.4% in females). Giacomini et al. reported a cohort of 34 patients with *CASK*-related disorders and reported that half of the patients suffered from epileptic syndromes. Among them, a main phenotype of epilepsy by *CASK* deficiency was late-onset drug-resistant spasms, some of which developed into developmental and epileptic encephalopathy [10]. It has also been reported that patients with *CASK*-related disorder can be affected by Ohtahara syndrome [6], West syndrome [9,62], absences epilepsy [5,11,14], Lennox-Gastaut syndrome [7], and myoclonic epilepsy [7,54]. Thus, epileptic phenotypes of *CASK*-related disorders are diverse among patients. Fifteen missense mutations have been identified in association with epileptic seizures, and the locations of these mutations do not converge on specific domains but diverge over all the functional domains of the CASK protein (Figure 3B). The wide distribution of missense mutations of *CASK* in the patients of epilepsy, which is also observed in the case of intellectual disability, indicates that epilepsy and intellectual disability may be due to multiple molecular mechanisms related to different functional domains of the CASK protein.

One of the CASK-partner molecules associated with infantile epilepsy is the NMDA receptor 2B subunit (GluN2B). Various mutations in the gene of GluN2B were found in patients suffering from infantile epilepsy [101,102]. The administration of NMDA has been used as a rodent model of infantile epilepsy [103,104,105]. The guanylate kinase domain at the C-terminal of CASK interacts with T-box transcription factor-1 (TBR1) and CASK regulates the gene expression of GluN2B in a TBR1-dependent manner [40,43]. We recently examined neuronal functional changes caused by the disruption of the CASK-TBR1-GluN2B pathway [92]. We first recorded cortical neurons of *CASK* heterozygous knockout mice and genotyped the recorded neurons using single-cell RT-PCR. We found that approximately 50% of neurons were CASK-positive, supporting a notion of the random distribution of CASK-expressing cells by XCI. Synaptic inputs to the CASK-expressing neurons in the heterozygous knockout mice were not different from those in the wild-type mice. On the other hand, the CASK-deficient neurons received more excitatory and fewer inhibitory synapses. The imbalance of excitatory and inhibitory synaptic inputs was due to the downregulation of GluN2B caused by the disruption of the CASK–TBR1 interaction. The increased excitability of neuronal circuits caused by the loss of *CASK* gene expression may explain the pathogenesis of epilepsy in patients with *CASK*-related disorders. The synaptic inputs to CASK-deficient neurons are stronger than those to CASK-expressing neurons, suggesting that CASK-deficient neuronal circuits are preferentially connected.

## 5. Unresolved Questions on the Pathophysiology of *CASK*-Related Disorders

Genetic studies of patients with genetic abnormalities in *CASK* and studies using *cask* knockout mice [106] are expected to help understand the pathogenesis of *CASK*-related disorders and to develop therapeutic strategies. However, to achieve this, some contradictions between human and animal-model studies must be resolved and understood in an integrated manner.

MICPCH syndrome is one of the pathogenic phenotypes of *CASK*-related disorders, for which there are differences between human phenotypes and mouse models. Magnetic resonance imaging (MRI) cerebellar morphology has been reported in approximately 50% of cases, with significantly smaller cerebellar volumes in both boys and girls, and some reports of cerebellar atrophy showed as much as 90% was abnormal [4]. In patients with *CASK*-related disorders, microcephaly has been shown to be progressive from the prenatal period [107]. On the other hand, with respect to studies in animal models, Najm et al. reported a reduction in cerebellar cortical thickness in *cask*-flox mice in which the expression of the *cask* gene decreased by about 20% (Supplementary Figure S5 in [4]). Mukherjee’s group has reported that the size of the cerebellum was 40% smaller in *cask* heterozygous knockout mice [79,92,94], which is still moderate compared with human cases. It has also been reported that the cerebellum size of *cask* heterozygous knockout mice is almost the same as that of the wild type during the first week after birth, after which the development of the cerebellum stagnates [95]. This cerebellar stagnation and atrophy may not be limited to early development; Patel et al. studied mice in which the *CASK* gene was deleted in most of the cells that make up the cerebellum [79]. In those mice, they reported a decrease in cerebellar size after 2 months of age, accompanied by the observation of cerebellar-dependent ataxia. These differences in the process of microcephaly and MICPCH between humans and mouse models are thought to be largely due to interspecies differences in neurodevelopment, and differences in the mutation patterns of the *CASK* gene. The lethality of the constitutive *cask* knockout has hindered the development of such studies. The introduction and analysis of these new methods are expected to provide answers to these questions.

No trend has been observed in pathogenic mutations of the *CASK* gene related to ID and epilepsy to date. Considering that each functional domain exerts a unique biological function by associating with different partner proteins, it is reasonable to assume that there are multiple molecular mechanisms underlying the ID or epilepsy in *CASK*-related disorders. Intellectual disability and epilepsy are known to be caused by genetic mutations in CASK partner proteins, including adhesion factors involved in synapse formation (e.g., Neurexin), presynaptic-release-related factors (voltage-gated calcium channels, SNARE-related molecules), and postsynaptic receptors (NMDA receptors) [108]. Interactions between CASK and the partner proteins must be examined to understand the mechanisms of *CASK*-associated intellectual disability and epilepsy and to develop new therapeutic strategies. Until recently, these protein–protein interactions were examined by biochemical assays targeting a few molecular mechanisms of interest. In addition to these classical approaches, the use of machine learning to predict higher-order protein structures, which has developed remarkably in recent years, is expected to dramatically improve our understanding of the protein–protein interactions underlying the pathogenesis of *CASK*-related disorders on a larger scale. To our knowledge, all CASK protein variants are speculated to be loss-of-function or hypomorphic variants. The improved prediction of protein structure and protein–protein interactions based on machine learning will aid in the discovery of unknown gain-of-function mutations that result in clinical phenotypes.

**Figure 4:**
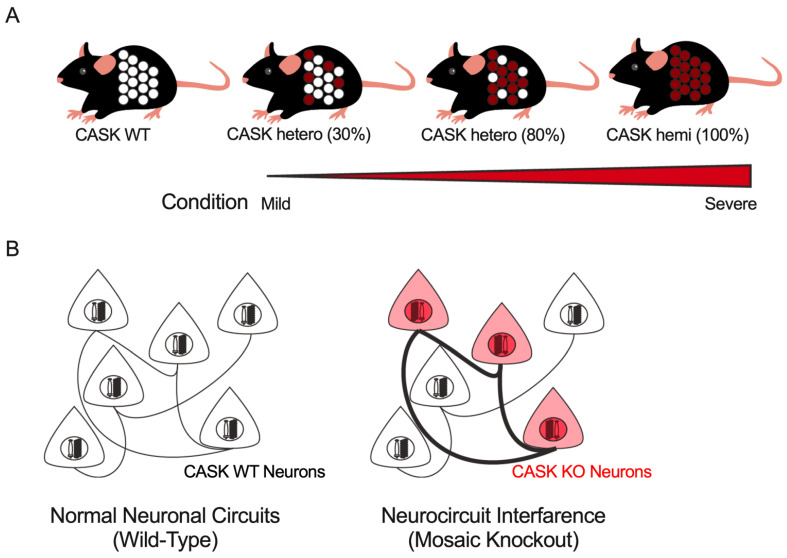
Mesoscopic hypotheses underlying the pathophysiology of *CASK*-related disorders as well as other X-linked neurological disorders in female patients. (**A**) All the somatic cells in male patients express a pathogenic mutant of the CASK protein (the rightmost), except for somatic mosaic cases of males. Female patients consist of a mixture of CASK-expressing (white) and -deficient (red) cells and the proportion of the two genotypes of cells may determine the severity of *CASK*-related disorders. (**B**) CASK-deficient neurons (red) are connected more preferentially to each other, and synaptic connections between CASK-expressing neurons are weaker. The “neurocircuit interference”, a genotype-independent neural circuitry, may explain the pathogenesis of *CASK*-related disorders.

In addition to *CASK*-related disorders, several other X-linked neurological diseases, such as *PCDH19*-related disorders [109], have been reported in which most patients were female. These diseases exhibit a range of clinical symptoms that vary in severity among individuals. One possible explanation for this variability is the proportion of gene-deficient neurons in an individual’s brain, which may be correlated with the severity of symptoms (Figure 4A). As previously discussed, epilepsy phenotypes were observed more frequently in males with *CASK*-related disorders than in females. This sexual difference in epilepsy incidence may be due to the proportion of CASK-expressing cells in the brain. It has also been demonstrated that *CASK*-related disorders may comprise relatively separate neuronal circuits with different physiological characteristics in a genotype-dependent manner. The cellular interference hypothesis, which proposes that interference between these two types of genetically distinct neuronal circuits results in the disruption of neuronal function, has been proposed to explain the pathophysiology of *PCDH19*-related disorders. This concept of neurocircuit interference may provide new insights into the neurocircuit mechanism of female-restricted intellectual disability and epilepsy in *CASK*-related disorders, as well as other X-linked disorders (Figure 4B).

To validate these hypotheses, it is necessary to simultaneously examine the pathology of the patient and the XCI pattern in the brain, but it is technically almost impossible to directly examine the XCI pattern in the patient’s brain. To address this challenge, researchers are turning to alternative approaches, such as utilizing mouse models that enable the simultaneous analysis of phenotypes and the brain XCI patterns. Such models offer a valuable tool to verify the hypotheses and contribute to the development of methods for estimating brain XCI patterns.

## 6. Conclusions

In this review, we have discussed significant associations between *CASK* gene mutation patterns and the pathophysiology of *CASK*-related disorders. Additionally, we described the molecular interactions that are necessary for understanding the molecular mechanisms of *CASK*-related disorders. Although we mainly focus on MICPCH and ID/epilepsy in this review, *CASK*-related disorders also include a wide range of clinical manifestations, including ophthalmologic conditions such as nystagmus, sensorineural hearing loss, short stature, and structural abnormalities of the heart, which were not covered in this review. Therefore, it is essential to elucidate the genetic background of these conditions through the further follow-up of patients with *CASK*-related disorders. Collaboration with the recently organized groups of patients with *CASK*-related disorders is expected to provide information on the pathogenesis of *CASK*-related disorders and the mode of genetic variation contributing to its development. Based on this comprehensive clinical information, we expect that an analysis of interactions with CASK using machine learning to predict the protein conformation and the generation of mouse models carrying specific genetic mutations from patients using genome editing will contribute to the development of future treatments for *CASK*-related disorders.

## Figures and Tables

**Table 1:** Genetic variants in the CASK protein on ClinVar database.

Mutations	Pathogenic	Likely Pathogenic	Benign ^1^	Uncertain Significance ^2^
Frameshift	30	6	0	1
Missense *	11	20	14	180
Nonsense	40	3	0	0

* Statistical differences were observed among the types of mutation (Chi-square test, *p* < 0.05). ^1^ Benign includes “likely benign” cases. ^2^ Cases of conflicting interpretations were included.

**Table 2:** Gender and phenotypes in *CASK*-related disorders.

Phenotypes	Gender	Severe	Mild/No	NA ^1^
Intellectual Disability	Male	58	4	3
	Female	124	5	3
Microcephaly/MICPCH	Male	35	11	19
	Female	107	15	10
Epilepsy *	Male	33	28	4
	Female	44	78	10

^1^ No clinical information available. * Statistical differences were observed between genders (Chi-square test, *p* < 0.05).

**Table 3:** Clinical phenotypes and mutations in *CASK*-related disorders.

Phenotypes	Mutations	Male	Female	Total
Intellectual disability **	Nonsense ^1^	24	109	133
	Missense	34	15	49
Microcephaly/MICPCH **	Nonsense ^1^	19	98	117
	Missense	14	9	23
Epileptic seizures **	Nonsense ^1^	16	40	56
	Missense	17	2	19
Ophthalmological anomalies *	Nonsense ^1^	16	38	64
	Missense	11	7	18

^1^ Cases of nonsense include frameshift mutations of the *CASK* gene and a micro-deletion of the X chromosome including the *CASK* gene locus. Statistical difference was observed between genders (Chi-square test, * *p* < 0.05, ** *p* < 0.0001).

## Data Availability

Not applicable.
